# The Developmental Process of the Growing Motile Ciliary Tip Region

**DOI:** 10.1038/s41598-018-26111-2

**Published:** 2018-05-22

**Authors:** Matthew J. Reynolds, Tanaporn Phetruen, Rebecca L. Fisher, Ke Chen, Brian T. Pentecost, George Gomez, Puey Ounjai, Haixin Sui

**Affiliations:** 1 Wadsworth Center, New York State Department of Health, Albany, NY 12201 USA; 2 Biology Department, University of Scranton, Scranton, PA 18510 USA; 3 Department of Biology, Faculty of Science, Mahidol University, Bangkok, 10400 Thailand; 4 Department of Biomedical Sciences, School of Public Health, University at Albany, Albany, NY 12201 USA

## Abstract

Eukaryotic motile cilia/flagella play vital roles in various physiological processes in mammals and some protists. Defects in cilia formation underlie multiple human disorders, known as ciliopathies. The detailed processes of cilia growth and development are still far from clear despite extensive studies. In this study, we characterized the process of cilium formation (ciliogenesis) by investigating the newly developed motile cilia of deciliated protists using complementary techniques in electron microscopy and image analysis. Our results demonstrated that the distal tip region of motile cilia exhibit progressive morphological changes as cilia develop. This developmental process is time-dependent and continues after growing cilia reach their full lengths. The structural analysis of growing ciliary tips revealed that B-tubules of axonemal microtubule doublets terminate far away from the tip end, which is led by the flagellar tip complex (FTC), demonstrating that the FTC might not directly mediate the fast turnover of intraflagellar transport (IFT).

## Introduction

Eukaryotic motile cilia/flagella are microtubule-based structures responsible for cellular locomotion and movement of extracellular fluids. In mammals, motile cilia are present in ciliated epithelial tissue of the airway, oviduct, or brain ependyma, and they serve as the tails of sperm cells. Defects in the cilium developmental process (or so-called ciliogenesis) are associated with medical disorders and dysfunction of related tissues, organs, and gametes^1–3^. Motile ciliogenesis has been studied for a half a century, primarily through investigations of cilium length and/or motility as cilia regrow following a deciliation event^4–7^. During development of the ciliary axoneme, microtubule bundles exhibit plus-end directed elongation at the ciliary tip region. The mechanism behind transportation of axonemal precursors to the ciliary tip was unclear until the discovery of intraflagellar transport (IFT)^8^. The current model for motile ciliogenesis involves a complex coordination of protein trafficking, protein production, and axonemal assembly^9,10^. Ciliated cells possess a pool of axonemal precursors localized beneath the cilium base^11^. These precursors can pass through the flagellar pore, and then be transported to the distal end of the cilium via anterograde IFT, which ensures a continuous growth event at the ciliary tip. Retrograde IFT is responsible for turnover of proteins at the distal ends of axonemal microtubule doublets (MTDs)^12,13^. Even after cilia reach full length, the IFT process and protein turnover continue at the ciliary tip. A dynamic balancing of the relative rates of anterograde and retrograde transport controls ciliary length^14,15^. A recent study determined that bi-directional transport on the same microtubule doublet is possible because motor proteins that transport IFT particles move in trains along different MTD sub-fibers; kinesin motors walk along the B-tubule, and dynein motors walk along the A-tubule^16^. It remains unknown whether this dynamic activity causes structural remodeling of the ciliary tip region during ciliogenesis and maintenance.

Although the processes by which axonemal precursors are transported to and from the ciliary tip region have been elucidated, the events of ciliary tip assembly and the transition from anterograde to retrograde IFT at the tip region remain unclear. The motile cilium tip has been studied, and its structure was diagrammed, based on results from negative staining and conventional electron microscopy (EM)^17^. There is an amalgamation of proteins at the tip of growing cilia known as the flagellar tip complex (FTC). The FTC remains enigmatic partially because few proteins within it have been identified. The proteins that have been identified include the microtubule end-binding protein EB1 and a 97-kDa protein otherwise found exclusively in kinetochores^18,19^. The FTC is proposed to play a role not only in regulation of ciliary beating but also in the transition between anterograde and the retrograde IFT^20^. There is evidence to suggest that IFT particles remodel at the cilium tip^13,21^. However, no evidence has been provided to support a direct interaction between the FTC and IFT trains, so whether IFT trains and the FTC interact to regulate IFT transition remains an open question.

In our pilot studies on cilia isolated from wild-type *Tetrahymena thermophila*, we noticed that isolated cilia displayed at least two distinct morphologies under negative staining EM. It motivated us to hypothesize that the morphological differences are related to various re-growth stages of the isolated cilia. In the work reported in this paper, we investigated the re-growing motile cilia from *Tetrahymena* using scanning electron microscopy and confirmed that the ciliary tip regions are subjected to morphological changes during ciliogenesis. We isolated re-growing motile cilia from deciliated *Tetrahymena* and *Chlamydomonas* at different time points, and studied their tip morphology by negative staining EM and a customized set of diameter profile analysis programs. Our results demonstrate that motile cilia tips display a dynamic, time-dependent morphology during re-growth. Cryo-electron tomography (cryo-ET) confirmed that these progressive differences are associated with the length difference between the central-pair microtubules and the growing ends of the MTDs.

## Results

### Tips of growing cilia display a morphological progression during cilia development

**Figure 1:**
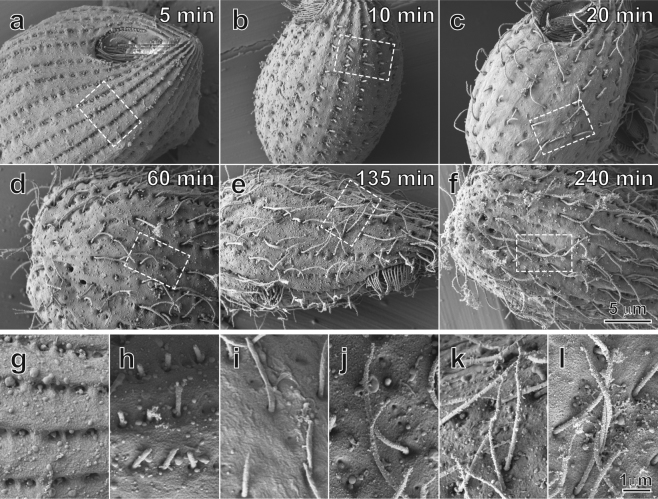
Scanning electron microscopy of re-growing motile cilia on deciliated *Tetrahymena* cells after different regrowth periods. Deciliated *Tetrahymena* cells after (**a**) 5 minutes, (**b**) 10 minutes, (**c**) 20 minutes, (**d**) 60 minutes, (**e**) 135 minutes, and (**f**) 240 minutes, respectively. The re-growing motile cilia in the local areas of (**a**–**f**), marked by the rectangles, have been enlarged and displayed in 5 minutes (**g**), 10 minutes (**h**), 20 minutes (**i**), 60 minutes (**j**), 135 minutes (**k**), and 240 minutes (**l**), respectively. The tip regions of these re-growing cilia display morphologies from relatively blunt (**g** and **h**), to beginning to protrude an extension (**i** and **j**), and reach a maximum at 135 minutes (**k**). Eventually, the lengths of the tip regions shorten and stabilize.

To investigate ciliary formation and development, *Tetrahymena* were deciliated using the dibucaine-HCl method, and cells recovering their cilia were examined at different time points using scanning electron microscopy. *Tetrahymena* cilia exhibited several distinct morphological features as they developed. During the initial phase of ciliary regrowth, cilia were typically less than two microns in length, and they had a hemi-capsule morphology as they emerged from the ciliary pocket (Fig. 1(a–c,g–i)). In agreement with previous studies^4^, somatic cilia regrew uniformly along rows following a complete deciliation event. There was also a period of rapid growth between 10 and 135 minutes.

As early as 20 minutes after deciliation, the distal ciliary region began to extend a pointed tip (Fig. 1c,i). This ciliary tip region extension coincided with elongation of the whole cilium (Fig. 1(d,e,j,k)). At later time points, ciliary tip regions exhibited shorter lengths (Fig. 1f,l). Proximal to this tip region, the cilia appeared to be of constant diameter. Taken together, these observations suggest that the tip region undergoes a consistent remodeling process as the cilium grows. However, resolution limits and variations in three-dimensional orientations of the cilia made it difficult to characterize this dynamic morphology systematically using scanning electron micrographs. We next expanded these studies using negative-staining transmission electron microscopy to view developing ciliary ultrastructure in greater detail.

### The ciliary tip diameter profile progressively transitions during cilium development

**Figure 2:**
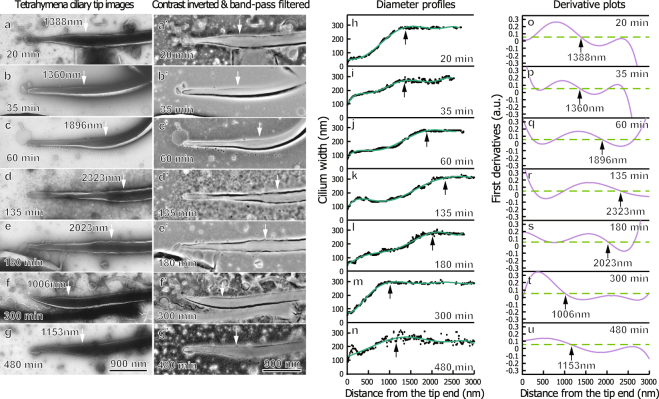
*Tetrahymena* ciliary tip regions remodel during cilia development. Negatively stained, re-growing cilia isolated from *Tetrahymena* at specified times are shown above. Electron micrographs of cilia isolated at various re-growth time before (**a**–**g**) and after (**a’**–**g’**) contrast inversion and band-pass filtering. The arrows indicate the end of a cilium’s tip region. (**h**–**n**) show the corresponding width profiles for each cilium and an interpolating best-fit curve. (**o**–**u**) show the first derivative plots of these curves. The tip region length is defined using the best-fit curve’s derivative and a cutoff slope at 0.052, corresponding to a 1.5° angle on both sides. The growing *Tetrahymena* ciliary tip region initially increases in length, until it reaches a maximum, then it decreases in length. Typically, cilia with longer tip regions had thinner diameters at the thinnest points.

We used negative-staining EM on re-growing cilia isolated from *Tetrahymena* (Fig. 2) to validate and further characterize this time-dependent morphological transition. The micrographs provided sufficient morphological detail for a quantitative description of ciliary ultrastructural features. Typical morphological features of the growing cilium include a thin diameter at the distal end, followed by a region of increasing diameter, until the cilium reaches full-width (Fig. 2a–g). It appears that the ciliary region intermediate to the absolute tip and the point of full width is an immature zone lacking the full complement of axonemal components. Portions distal to the mature regions are considered part of the ciliary tip region in our analysis.

We designed a systematic method to define the transition point between the immature zone and the mature regions objectively. This was accomplished using a set of customized Java programs as a plug-in package for ImageJ^22^, as briefly described in the method section. Using this method, the length of the ciliary tip region can be defined and the progress of the tip growth can be described based on the length of this region. An explanation of the theory and implementation of this method is summarized in the supplemental text.

Figure 2 shows representative isolated cilia (Fig. 2a–g), along with their processed images (Fig. 2a’–g’), diameter profiles (Fig. 2h–n), and derivative plots (Fig. 2o–u). The arrows in panels indicate the end of the ciliary tip region, as defined above. This quantitative measure clearly showed that the length of the ciliary tip region changes over the 8 h regrowth period. In the beginning of cilia growth, the length of the ciliary tip region elongated and reached its maximum length after around 135 minutes of regrowth (Fig. 2d). After this maximum length was reached, the ciliary tip region slowly decreased in length until the cilia exhibit short-tipped morphologies (Fig. 2f,g). This growth process took place over the course of several hours, with the stable, short-tipped morphologies being present around 5 hours after the initial deciliation.

From these profiles, trends in tip diameter and length could be ascertained. In general, cilia with the longest tips (such as those shown at 60, 135, and 180 minutes) had very thin extensions (Fig. 2c–e); oftentimes cilia with tip regions around two microns had diameters as narrow as 75 nm. In ciliary tip regions with these very narrow extensions, a bulging of the very distal portion was occasionally visible, apparently due to the FTC. Meanwhile, cilia with shorter tip regions (such as those shown at 35 and 300 minutes) typically displayed wider, though still less than full-width, tip regions. In agreement with typical ciliary diameters, all cilia had full diameters between 200 nm and 300 nm, suggesting that negative staining produced minimal artifacts affecting ciliary diameter. In all of the isolated cilia, the FTC was clearly seen, indicating that the flagellar tip complex assembles at the distal ciliary tip region within 20 minutes of ciliary re-growth, and that the FTC persists throughout the cilium’s lifetime. Lastly, many ciliary diameter profiles exhibited a stepped appearance, which may be due to uneven termination of axonemal MTDs in the tip region.

In order to determine whether these observed morphological characteristics of regenerating *Tetrahymena* cilia are conserved among protists, isolation and width profile determination experiments were replicated using re-growing cilia from *Chlamydomonas reinhardtii*, as shown in (Fig. S1). Overall, *Chlamydomonas* exhibited a similar trend of the ciliary tip region growing in length before decreasing in length. However, *Chlamydomonas* cilia did not typically exhibit diameters below 100 nm, and the tip regions were typically less than 20% of the total ciliary length. Therefore, *Chlamydomonas* cilia exhibit a similar, though less dramatic, tip region re-growth trend.

### Development of the ciliary tip region continues after the cilium reaches full length

Previous investigations into ciliogenesis primarily studied length of the entire cilium during the elongation phase and/or cell motility^4,6,23–25^. In order to consider tip region development in the context of the growing cilium’s length, we determined both the average ciliary tip length and the average length of the entire cilium at different times of regrowth.

**Figure 3:**
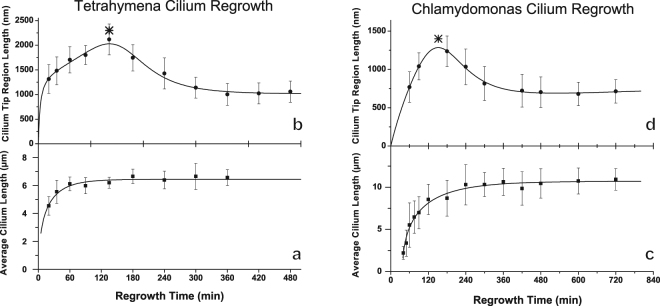
Maturation of the ciliary tip region continues after whole cilia reach full length. Plots (**a** and **c**) display the mean full lengths of growing cilia, with error bars representing standard deviation, which extend rapidly during the first phase of growth, reaching a maximum length of approximately 6.5 microns for *Tetrahymena* (after about two hours) and 10.7 microns for *Chlamydomonas* (after about four hours), respectively. Plots (**b** and **d**) show the mean tip region lengths, with error bars representing standard deviation, of growing cilia measured using our objective method. In both species, the ciliary tip region initially increases in length, with a maximum of 2020 nm at 135 minutes in *Tetrahymena* and 1280 nm at 150 minutes in *Chlamydomonas* (as denoted with an asterisk) before decreasing in length. The tip region length asymptotically decreases to 1070 nm for *Tetrahymena* and 680 nm for *Chlamydomonas*. Figure S2 displays the ratio of ciliary tip region length to whole cilium length, which demonstrates that the length percentile of the ciliary region reaches a peak at a growth time of about 135 min in both *Tetrahymena* and *Chlamydomonas*. Notably, the maturation of the ciliary tip region continues for over five hours following deciliation, whereas the whole cilium reaches full-length around two hours after deciliation.

The method described above was used to measure the average length of the ciliary tip region from at least 27 cilia isolated at different time points for each cell type. As shown in Fig. 3, the ciliary tip regions of both *Tetrahymena* and *Chlamydomonas* initially extended outwards reaching a maximum length of over 2 microns at 135 minutes for *Tetrahymena* (Fig. 3a) and a maximum length of 1.3 microns at 150 minutes for *Chlamydomonas* (Fig. 3c). After the tip region reached its maximum length, it decreased in length until it asymptotically approaches 1070 nm in *Tetrahymena* and 680 nm in *Chlamydomonas*. Based on the rate of change, in both species, it took approximately six hours to reach full, mature tip length. The results of these flagellar regeneration kinetics experiments on the full length of both *Tetrahymena* and *Chlamydomonas* cilia closely followed previously reported trends^6,23^. Based on their respective trend profiles, *Tetrahymena* cilia reached a full length of 6.5 microns, while *Chlamydomonas* cilia reached a full length of 10.7 microns.

Comparing the length profiles for the full length of the whole re-growing cilia (Fig. 3b,d) to that of the tip region (Fig. 3a,c), the whole cilia display a fast extension phase initially, followed by a plateau growth phase with minimum elongation of the full length. In contrast, the tip region development and maturation continued long after the cilia had reached full length. It took approximately six hours for the ciliary tip region to establish a stable length. During the initial, fast extension phase, the ciliary tip region made up approximately one third of the total cilium length (Fig. S2). This proportion decreased non-monotonically until stabilizing at a species-dependent proportion. The time scale of this process was much longer than polymerization rates of tubulin on MTDs^26^.

### Structural features of the growing cilium tip

Conventional EM has revealed the tip structure of mature *Tetrahymena* cilia^17,27,28^, in which the longest portion is the central pair microtubule singlets capped by a ball-shaped FTC resembling a post-light, and the central pair complex is surrounded by 9 microtubule doublets forming a cylindrical configuration. At the distal ends of the microtubule doublets, the complete A-tubule extends farther and is stabilized by a globular protein complex that links to the ciliary membrane by fibrous proteins known as distal filaments. We performed a cryo-EM study on the cilium tips of re-growing cilia to better understand the tip structure in the regrowth phase. Our EM study demonstrates that the overall structure of the growing tip is largely similar to the architecture of the mature cilium tip; however, the more proximal portion of the ciliary tip region differs from the mature cilium. The growing axoneme’s most distal components are two microtubule singlets of the central pair complex and a FTC that caps the central pair microtubule singlets together.

**Figure 4:**
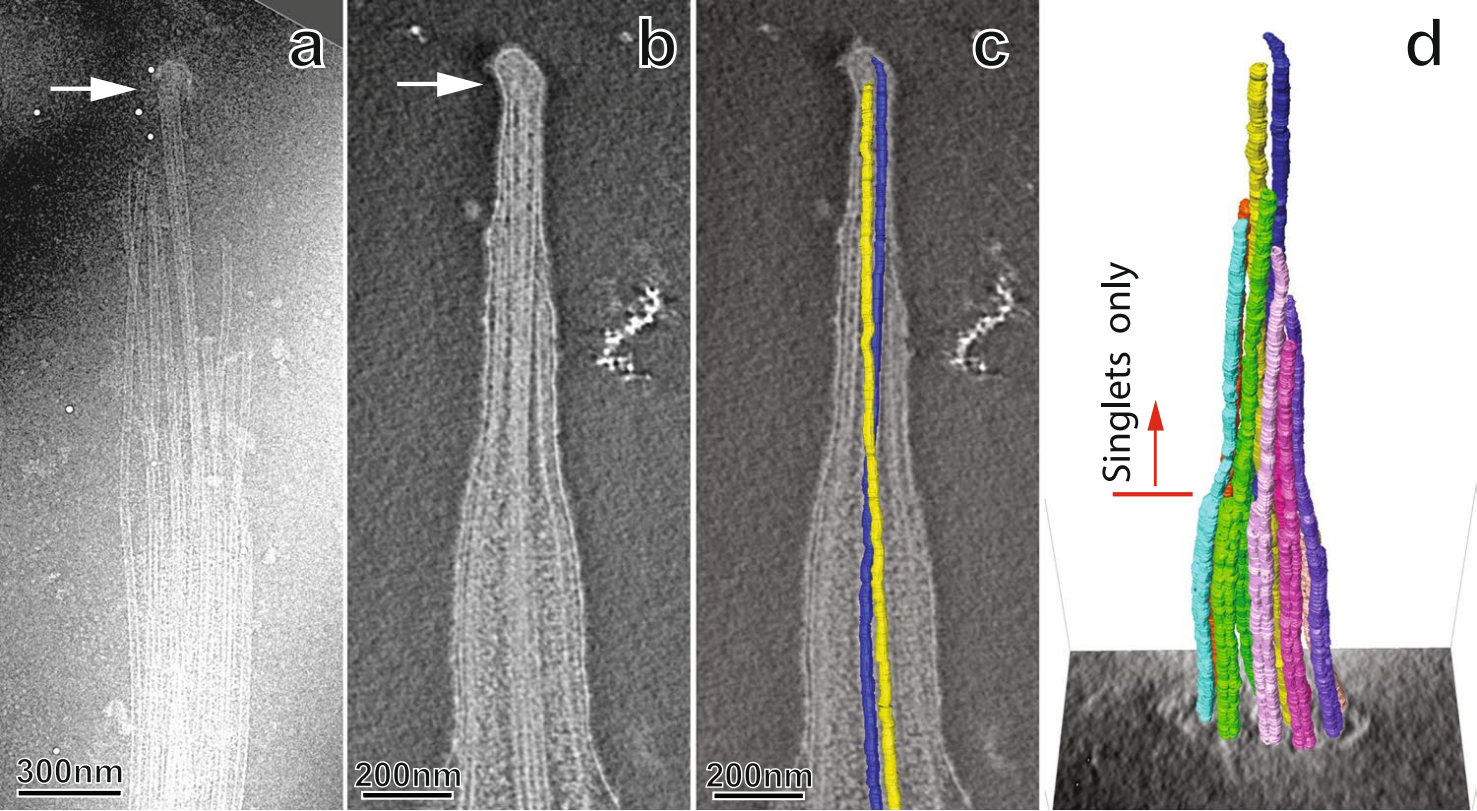
The cryo-electron microscopy of growing ciliary tip region after re-growth for 180 minutes. A micrograph of a splayed ciliary tip region, shown in (**a**), suggests that the distal axoneme lacks standard axonemal components. Tomograms of the ciliary tip region, shown in (**b**) (a central slice) and (**c**) **(with added model of the central pair)**, confirm that the FTC sits on the central pair, and the central pair is physically constrained by membrane. The central pair twists, is outlined by the model microtubules in blue and yellow in (**c**). The MTD A-tubules extend into the ciliary tip region at least 400 nm farther than the B-tubules do. The distal tip therefore composes only microtubule singlets as marked by the red arrow in (**d**). These extended A-tubules are not decorated with axonemal spacer proteins.

Previous studies have used splayed cilia as indications of axonemal composition in the tip region^29^, and during specimen preparation, isolated cilia occasionally displayed membrane damage localized to the tip region. This resulted in a splaying of the tip region’s contents, while the remainder of the cilium remained intact. These frayed tip regions allowed for easier visualization of the ciliary tip region’s components. Figure 4a shows that the zone approximately 50 to 400 nm proximal to the FTC is occupied by the central pair microtubules and extended singlet A-tubules. Notably, the A-tubules extended into the ciliary tip region appear undecorated, whereas axonemal spacer proteins (radial spokes and outer dynein arms) are usually visible under cryo-EM^30–33^. This observation, along with the absence of B-tubules suggests that the growing ciliary tip region lacks a full complement of axonemal proteins.

While splayed ciliary tip regions provide clear visualization of the ciliary tip region’s contents, it is possible that these features could be artifacts caused by membrane disruption. A more detailed, native state of the growing ciliary tip region was achieved via cryo-ET. Tomograms of growing cilia allowed us to verify that the immature ciliary tip region lacks well-organized spacer proteins like those of the mature axoneme, and the FTC is positioned in the most distal region of the cilium, as marked by the arrow in the longitudinal central section of a cryo-tomogram of a 3 h-regrowth cilium tip (Fig. 4b). At the tip of the growing cilium, the FTC-capped central pair is surrounded by a set of long microtubule singlets, not the standard nine doublet microtubules. These singlets are extensions of the MTD’s A-tubule. These microtubule singlets are visible in the tomogram (Fig. 4b), and they are clearly shown in the frayed tip (Fig. 4a). In the tomogram of Fig. 4b, the B-tubules are separated from the tip end, where the FTC is located, by over half a micrometer (ranging from 511 nm to 1,029 nm). In addition, we observed that the central pair has a left-handed twist as shown in the model maps in Fig. 4c.

## Discussion

In this study, we identified and characterized the growth process of the motile ciliary tip. Mechanistic similarities in the growth process of the ciliary tip were found in both *T. thermophila* and *C. reinhardtii*, indicating that this process is not species specific. Although the regeneration kinetics of ciliary growth calculated using the full-length of isolated cilia from both *Tetrahymena* and *Chlamydomonas* are consistent with previously modelled regeneration kinetics^15^, in our observation, ciliogenesis contains two main steps: an initial step where the length of the full cilia undergoes rapid extension, followed by a step of plateau phase growth, in which cilium length remains almost constant but the tip region continues to develop and mature. Interestingly, the morphology of the tip region of growing motile cilia exhibits a characteristic progression. During the rapid growth, cilia appear as hemi-capsules of short length, which is consistent with previous studies on the early regrowth of *C. reinhardtii* cilia^6^. It is likely that the FTC might have not fully assembled at this point. The tip region of motile cilia then undergoes a progressive structural modification, which involves an extension of the central pair microtubules followed by extension of the A-tubules of MTDs and eventually the B-tubules, on the scale of hours. To gain a better understanding of the assembly characteristics, we developed a set of user-friendly programs to measure the length of the tip region from negative stained images of growing cilia. Diameter profiles and lengths of each ciliary tip region were determined. In the negatively stained micrographs, we can clearly see contrast from individual MTDs, but it is difficult to identify how many there are. The diameter profiles allow us to determine that these cilia tip regions may be as narrow as 60 nm: an enclosed space that can accommodate either only one MTD or just the central pair. This raised the question of whether the central pair or a MTD projects forward. This question is answered by our cryo-EM study, which showed that indeed the central pair, not a MTD, leads cilium growth at the tip region.

**Figure 5:**
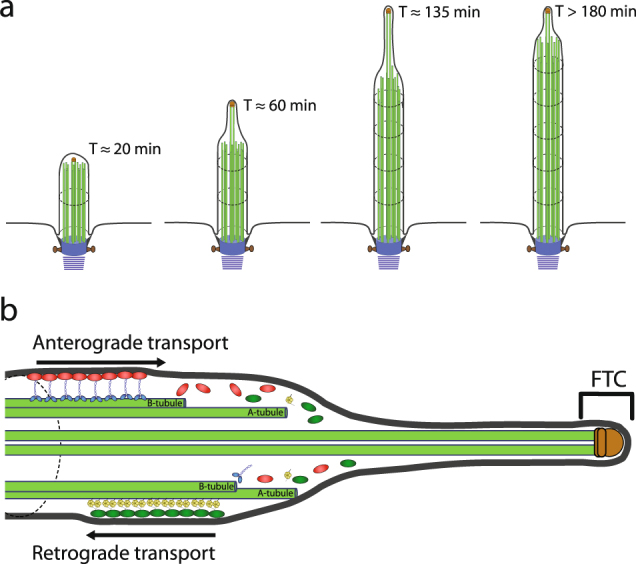
Schematic summary of cilium re-growth following amputation. The previously unidentified morphological transition of re-growing cilia is diagrammed in (**a**) (with Adobe Illustrator). First, the re-growing cilium extends from the ciliary pit with a round-tipped morphology (T ∼ 20 min), and the MTDs and central pair microtubule singlets are all approximately the same length. Then, when the cilium rapidly grows, the central pair microtubule singlets, capped by the FTC extend farther than the neighboring MTDs (T ∼ 60 min and 135 min). After the cilium reaches full length at T ∼ 135 min, the ciliary tip region continues to remodel as the distal axoneme assembles. The proposed molecular mechanism of distal axoneme assembly is shown in (**b**). In this model, anterograde IFT trains dissociate at the MTD B-tubule terminus several hundred nanometers proximal to the FTC and retrograde IFT train assembly occurs on the MTD A-tubules.

Although cilia continue to exhibit dynamic turnover after they have already reached full length, previous investigations of motile ciliogenesis often measured the full length of cilia, and they generally considered cilia mature once they reach the slow elongation phase^14,15^. Our data, however, suggest that the cilia continue to mature several hours after they reach full length. Therefore, the slow elongation phase is not just a period of protein turnover and overall length maintenance, but it is also a period of continued maturation process in the tip region. The overall morphologic changes and the underlying changes in ciliary architecture are diagrammed in Fig. 5a. These investigations give insights into organelle size regulation and maturation, because throughout the process of tip region development, the overall length of a cilium is very well maintained at an essentially constant length.

Although the architecture of the growing motile ciliary tip region is largely similar to the mature axoneme, our cryo-ET investigations revealed important distinctions in composition and structure. It is well established that the motile ciliary tip region contains the FTC, which is composed of several proteins not found in the mature axoneme^18,19^. The FTC-capped central pair microtubules appear to push the membrane at the tip of the regenerating cilium. The capped central pair reaches the full length before other axonemal microtubules. It is also clear that the elongated A-tubules of MTDs do not extend beyond the central pair tip. This makes the FTC-capped central pair microtubule the defining factor or the controller of the maximum length of cilia. In our studies, the central pair complexes of the *Tetrahymena* cilia display a left-handed twist, in agreement with previous reports that bend propagation during the ciliary beat drives twisting of the central pair in *Chlamydomonas reinhardtii* and *Paramecium tetraurelia*^34,35^. Furthermore, our cryo-tomographic study of the membrane-intact, isolated *Tetrahymena thermophila* cilia, demonstrates that central pair complex twisting is also present in the tip regions where radial spokes are not mature. This result supports the conclusion that the twist does not depend on the interactions between radial spoke heads and the central pair^35^.

Our structural investigations from cryo-EM of splayed ciliary tip regions and cryo-ET of membrane-intact ciliary tip regions of regenerating cilia demonstrated that the growing ciliary tip region lacks the full complement of proteins found in the mature axoneme. These differences have consequences for both ciliary assembly and IFT train turnaround. The immature ciliary tip region is composed of microtubule singlets, which are long extensions of the A-tubules from axonemal MTDs. Such structural features provide important insights into cilium assembly that involves IFT system. It has been well established that IFT serves as the primary mechanism for protein transportation across the base and distal ends of the cilium during both cilium assembly and maintenance. In growing cilia, particularly in the fast-growing stage (the first two hours in Fig. 3), the central pair, capped by the FTC, can be over a micrometer from the distal end of the B-tubules of MTDs. We know that anterograde IFT trains rely on kinesin motors walking along the B-tubule of the MTD^16^. Our data indicate that in the growing cilium, the normal anterograde IFT may stop at where the B-tubules terminates (marked by red arrow in Fig. 4d), far away from the ciliary tip end. Meanwhile, the central pair and the A-tubules are tightly wrapped by ciliary membrane as shown in Fig. 4b. This means there is limited room for a fast diffusion of large IFT complexes to the very tip of the growing cilium where the FTC caps the central pair microtubules. Therefore, it is unlikely that the FTC would play major roles in IFT reorganization and turnover, in contrast to a previously suggested model^20^. The proposed molecular mechanisms of distal axonemal assembly that are most plausible in the context of our tomographic studies are diagrammed in Fig. 5b.

Diffusion may be required for precursor proteins to reach the growing tip end of the extending central pair and the A-tubules of MTDs because anterograde IFT does not directly reach the growing tip ends where the extension happens for central pair microtubules and singlet A-tubules (Fig. 4d). However, the small, restricted space available, due to the tightly wrapped ciliary membrane around the central pair, does not favor a free fast diffusion of precursor proteins. Therefore, it is unclear how precursor proteins reach the growing tips of the central pair complex and the A-tubules of the doublet microtubules.

At the growing tip regions, both the central pairs and the A-tubule are singlet microtubules with tubulin as the primary component protein. If their extension is simply limited by the available tubulins that reach the tip region, the singlet A-tubules and the central pair should be of a similar length. However, our data have demonstrated that central pair microtubules are always longest at the growing ciliary tip with a significant length difference from that of the A-tubules. It could also be possible that the FTC plays a role in promoting central pair microtubule extension. However, the microtubule growth at the tip is not a simple extension limited by availability of tubulins. The time required for ciliary tip region maturation is on the scale of hours, which also suggests that tip region maturation is not limited by microtubule-doublet polymerization kinetics^26^. The nature of factors limiting the ciliary tip maturation process is unclear. Likely factors include post-translational modification of the microtubule complexes^36,37^, and/or the availability of MTD-specific intralumenal microtubule associated proteins^38–41^.

In this study, we discovered that tip regions of growing motile cilia are subject to a process of structural remodelling and maturation, which completes long after a cilium reaches its mature length. This new finding is not species specific, and therefore it is an important complementary knowledge to what we have known about cilia generation and maturation. Using a customized set of programs, we analyzed the morphologies of growing cilia tips at different growth times and clearly revealed the trend of ciliary tip growth and maturation. Cryo-electron tomographic studies of the growing ciliary tips determined that the growing ends of MTD B-tubules are significantly far away from the tip end led by the FTC. The trend profiles and the structural information from our electron tomographic study provided important insights into the relationship between IFT and FTC in ciliogenesis and the maintenance of cilia length.

## Materials and Methods

### Cell culture

Standard *Tetrahymena* and *Chlamydomonas* cell culture conditions were followed to maintain cell lines and to prepare cells for deciliation. Room-temperature stock cultures of the non-exocytozing *Tetrahymena thermophila* strain SB711, a gift from Prof. Eduardo Orias, were grown in modified Neff’s media. To prepare cells for cilia isolation, stock culture cells were inoculated in SSP media and allowed to grow for 24 h at 30 °C, with shaking. The volume of SSP media was then increased eight-fold and cells were cultured for another 24 h under these conditions. Cells were then spun down at 700x*g* and then transferred to an equal volume of starvation media (10 mM Tris-HCl, pH = 7.5) and cultured for 18 to 20 h before deciliation.

*Chlamydomonas reinhardtii* algae, strain CC-137+, a gift from Prof. George Witman, were cultured in TAP media at room temperature. A light/dark cycle of 18/6 h was used. Cells were grown to approximately 1 × 10^6^ cells/ml, and approximately 150 ml of cells were used for each deciliation.

### Tetrahymena cilia isolation

To ensure that cilia from the same sample were at the same stage of growth, an initial deciliation was performed. It was determined through pilot studies that cells deciliate most completely using a modified version of the dibucaine method^42^. Briefly, cells were spun down at 600x*g* for 7 minutes and re-suspended in half of their growth volume of starvation media, and dibucaine-HCl was added to a concentration of 300 µM for a 5-minute incubation. Then, fresh starvation media was added to double the volume immediately before pelleting cells at 1200x*g* for 5 minutes. The deciliated cells were suspended in the original volume of fresh starvation media for the regrowth period. After allowing the cilia to regrow for a specified time between 35 minutes and 6 h, the second deciliation was performed. The second deciliation was identical to the first deciliation, except the dibucaine concentration was 450 µM. After the 5-minute dibucaine-HCl incubation, glutaraldehyde was added to 0.025% and EGTA was added to 0.5 mM for a 30 s incubation. Then, an equal volume of 4% sucrose in ice-cold starvation media was added to dilute the dibucaine, and all subsequent steps were performed at 4 °C. The sample was centrifuged at 1550x*g* for 7 minutes to pellet cell bodies. The supernatant was recovered and centrifuged at 1550x*g* for 7 minutes again. The supernatant was recovered and centrifuged at 17,000x*g* for 40 minutes to pellet cilia. The final pellet containing isolated cilia was suspended in a minimal volume of starvation media for plunge-freezing.

### Chlamydomonas flagella isolation

*Chlamydomonas* cultures could not survive the dibucaine deciliation method, so the pH-shock method was employed for the first deciliation^43^. Briefly, cells were centrifuged at 600x*g* for 5 minutes and re-suspended in one-tenth their growth volume in fresh TAP media, and acetic acid was used to rapidly drop the pH from neutral to 4.5 for 60 s. Then, the media’s pH was rapidly neutralized with aqueous KOH. Fresh TAP media was added to double the volume, and cells were pelleted before resuspension in fresh TAP media. After allowing the cilia to regrow for a specified time between 35 minutes and 3 h, the second deciliation was performed using the dibucaine method, with the following modifications. The cells were concentrated to one-eighth the original volume in modified HMES buffer (10 mM HEPES, 5 mM MgSO_4_, 0.5 mM EGTA, and 2% sucrose, pH = 7.4), plus 0.025% glutaraldehyde, and 625 µM dibucaine was used for deciliation. The cilia were isolated in the same manner as the *Tetrahymena*, except the final centrifugation was at 31,000x*g* for 30 minutes.

### Negative staining

Preparation of isolated cilia for negative staining TEM observation was carried out using 400-mesh copper grids with a thick, continuous carbon coating that had been glow-discharged for 60 s immediately before sample deposition. A 3.0 µl aliquot of specimen was applied to the grid for 90 s. The specimen was blotted, and 1% uranyl acetate was applied to the grid for 90 s. The grid was then blotted and allowed to dry for at least 1 h before transferring into a JEOL-JEM1400 electron microscope operating at 100 keV. For each of the areas of interest, the z-height was adjusted to eucentric position before taking images to ensure magnification accuracy. Micrographs were taken at 8000X magnification and were analyzed using ImageJ.

### Analysis of ciliary tip region diameter

The analysis of the tip region of isolated cilia in negatively stained micrographs was performed using a set of custom-written Java programs. A detailed description is provided in the supplementary material. Briefly, micrographs were contrast-inverted and then bandpass-filtered to enhance the cilium’s edge contrast. Then, a polyline was drawn along the central axis of each cilium, starting from the absolute tip, and a width-determination program generated a diameter profile. An interpolating polynomial curve of degree six was fit to the diameter profile, and the location where the slope decreased below 0.0532 (equivalent to a 1.5° incline on each side) is used to define the position separating the mature full diameter region with the cilium tip region of an increasing diameter (shown as the arrows in Fig. 2). Then potential lengths were reported. From these potential lengths, the cilium’s tip region length was measured by determining which potential length agreed with the original micrograph. Parameters for the best-fit curves were determined using the nonlinear least-squares Marquardt-Levenberg algorithm implemented in Gnuplot. With the above strategy, a set of Java programs were written as a plug-in package for ImageJ^22^ and used for this analysis of approximately 900 electron micrographs of cilia. The diagrams of the tip architecture changes during cilium growth, shown in Fig. 5, were prepared using Adobe Illustrator (Adobe Systems In., San Jose, CA).

### Characterization of growing cilia and ciliary tip regions

In order to characterize ciliary development using the length of the entire cilium and the length of the tip region, average lengths of these features were plotted as functions of time. The ciliary tip region measurements appeared to exhibit the following characteristics: the ciliary tip region started at length zero, and it then sequentially reached a local maximum, an inflection point, and asymptotically approached a final length. Based on these conditions, a curve of best fit was determined using a quotient of two trinomials of the form (ax^3^ + bx^2^ + cx)/(dx^3^ + ex^2^ + fx + g). From these curves, the local maxima and horizontal asymptotes were determined. In order to characterize the length of the entire cilium as a function of time, we fit our data to the solution to the differential equation described in^15^.

### Cryo-EM sample preparation

Preparation of isolated cilia for cryo-EM observation was carried out using 300-mesh copper grids with a thin, continuous carbon coating that had been glow-discharged for 30 s immediately before sample preparation. A 2.5 µl aliquot of 0.1% poly-L-lysine (w/v in water) was applied to the grid for 1 minute. The grid was blotted, and subsequently washed twice in water. Then, water was applied to the grid a third time, and the grid was loaded into the Gatan CryoPlunger3. The grid was blotted, and a 2.5 µl aliquot of cilia specimen that had been mixed 3:1 with 15 nm gold particles was applied to it for 20 s. Then, single-side blotting was used to blot the grid for 6.0 s before plunging into liquid ethane at −174 °C. The frozen grids were stored in liquid nitrogen until loaded into a JEOL-JEM3200FSC/PP electron microscope. Data were acquired using either a CCD camera or a Gatan K2 Summit direct-electron detector.

### Cryo-ET data collection and processing

All electron tomographic tilt series data sets were collected using SerialEM^44^ with a tilt increment of 2 or 3 degrees. The JEM-3200FSC was operated at 300 keV with zero-loss energy filtering, slit width 24 eV. The pixel size on the specimen was 0.36 nm and the total electron dose was about 70 e^−^/A^2^. Images were recorded at a target underfocus between 4 and 8 µm on a K2 Summit direct-electron-detection camera (Gatan, Pleasanton, CA) with 5 to 8 frames per second during an exposure time of 1 to 1.5 sec. Frame alignment was carried out with the “Unblur & Summovie” package^45–47^. Tomographic reconstruction were carried out using Etomo/IMOD^48^. Bilateral filtering was applied to denoise the reconstructed tomograms with functions provided in EMAN2 package^49,50^.

### Scanning electron microscopy

Tetrahymena samples collected from different time points of regrowth after deciliation were fixed by 1% glutaraldehyde and placed in pre-processed aluminum carriers from the M. Wohlwend GmbH, Engineering office. Before adding *Tetrahymena* cells, the carriers were carbon coated and glow discharged (two minutes) followed by treatment of poly-L-lysine coating to increase cell attachment. The *Tetrahymena* cells in the carriers were subjected to a six-step gradient dehydration of ethyl alcohol (10%, 20%, 40%, 70%, 90%, and 100%) followed by critical-point drying using Samdri-795 with a 15 minutes purging process. Afterwards, the samples in the carrier were fixed on conductive stubs with silver glue and gold-coated for 45 seconds using a CRESSINGTON Sputter Coater. SEM images were captured at 2 kx, 5 kx, and 10 kx magnifications using Secondary Electrons Secondary Ions detector of a ZEISS NEON 40EsB High Resolution Dual Beam Scanning Electron Microscope.

## Electronic supplementary material

Supplementary Materials

Custom tools for image analysis
