# Supplementary material for: The Developmental Process of the Growing Motile Ciliary Tip Region
Source: Sci Rep. 2018 May 22;8:7977. doi: 10.1038/s41598-018-26111-2 (PMC5964098; doi:10.1038/s41598-018-26111-2)

The Developmental Process of the Growing Motile Ciliary Tip Region

Matthew J. Reynolds[1,2], Tanaporn Phetruen[1,3], Rebecca L. Fisher[1], Ke Chen[1], Brian T. Pentecost[1], George Gomez[2], Puey Ounjai[3], Haixin Sui[1,4,*]

1. Wadsworth Center, New York State Department of Health, Albany, NY 12201, USA
2. Biology Department, University of Scranton, Scranton, PA 18510, USA
3. Department of Biology, Faculty of Science, Mahidol University, Bangkok 10400, Thailand
4. Department of Biomedical Sciences, School of Public Health, University at Albany, Albany, NY 12201, USA

* Author for correspondence: P.O. Box 509, Albany, NY 12201-0509; Phone: +1-518-474-4235
E-mail: haixin.sui@health.ny.gov

Running title: Ciliary tip development

Key words: ciliogenesis, ciliary tip, flagellar tip complex

Supplemental Figures:

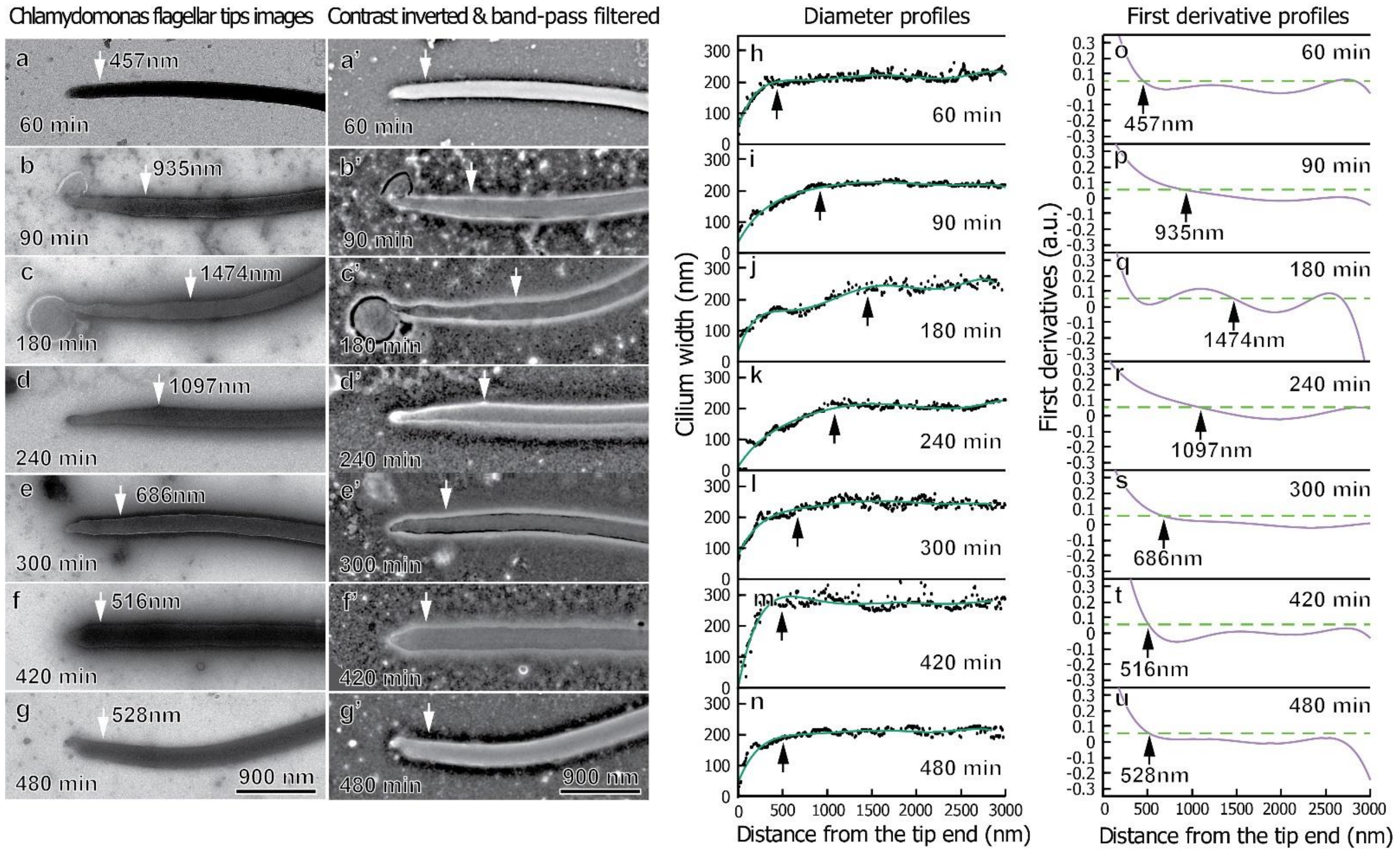

Figure S1: The diameter profile trends of the developing cilium are conserved among protists. Negatively-stained, re-growing cilia isolated from *Chlamydomonas* at specified times are shown above. Electron micrographs of isolated cilia at various re-growth time before (**Fig. S1a -S1g**) and after (**Fig. S1a' -S1g'**) contrast inversion and band-pass filtering. The arrows indicate the end of a cilium's tip region. **Fig. S1h – S1n** show the corresponding width profiles for each cilium and an interpolating best-fit curve. **Fig. S1o -S1u** show the first derivative plots of these curves. The tip region length is defined using the same criteria as for the *Tetrahymena*. The developing *Chlamydomonas* ciliary tip region initially increases in length, until it reaches a maximum, then it decreases in length.

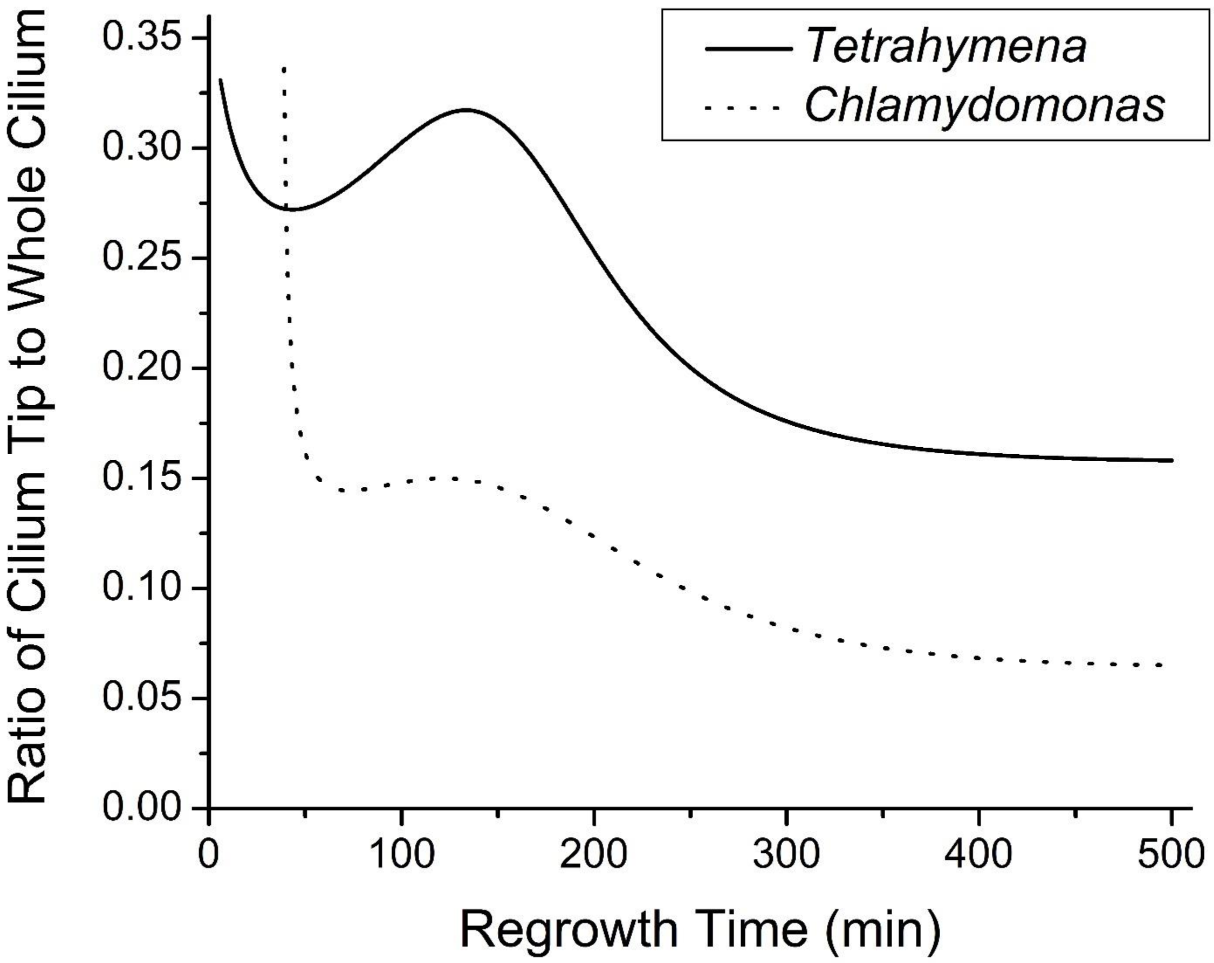

Figure S2: Ratio of ciliary tip region length to whole cilium length decreases non-monotonically as cilia re-grow in both *Tetrahymena* and *Chlamydomonas*. The ratios of the ciliary tip to the length of the whole cilium, based on the regrowth trends in **Fig. 3**, are plotted. In both species, there is a local maximum at approximately 135 min, coinciding with the maximum tip region length. This is followed by an asymptotic decrease to a ratio that is species-dependent.

Supplemental document:

Instruction of the custom modules as ImageJ plus-ins for ciliary tip morphology analysis

Description of Algorithm for Width Profile Determination:

In order to generate diameter profiles, images of negatively stained cilia were processed so that the custom edge-detection algorithm performed optimally. The images were first processed with a median filter. Then, a bandpass filter was used to preserve ciliary edge contrast. The contrast of the image was inverted so that the contrast from each edge would be detectable as maxima. A user then drew a polyline along each cilium's central axis, and the FlagellumWidthProfile plugin performed edge-detection as follows. Line segments of width one pixel were extended perpendicularly to the polyline, and edges were detected using an exhaustive, peak-pairing optimization algorithm. The algorithm found local pixel intensity maxima on each side of the polyline; then each peak was exhaustively paired with peaks from the other side, and their relative fitness as a peak pair was evaluated using an objective function. The objective function evaluated peak pairs based on their displacement of their midpoint from the polyline and their individual brightness; peaks with a higher brightness had higher fitness, and peak pairings with a midpoint closer to the polyline had higher fitness. The peak pair with the highest fitness was obtained pixel-wise along the polyline, and if it had a sufficiently high fitness, then it was considered to be successfully identified. For each pair of peaks that the algorithm successfully identified, the distance between the edges and distance along the cilium's central axis from the absolute cilium tip were recorded. Using Gnuplot, these data were plotted as a scatterplot along with a sixth-degree polynomial curve of best fit for tip region length determination.

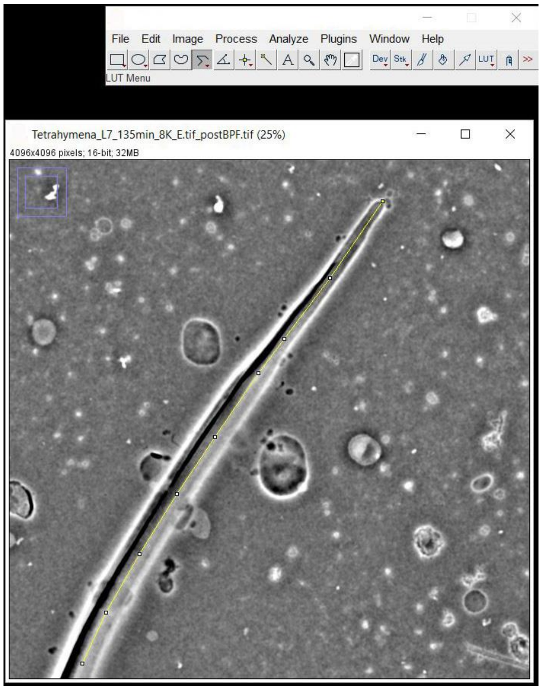

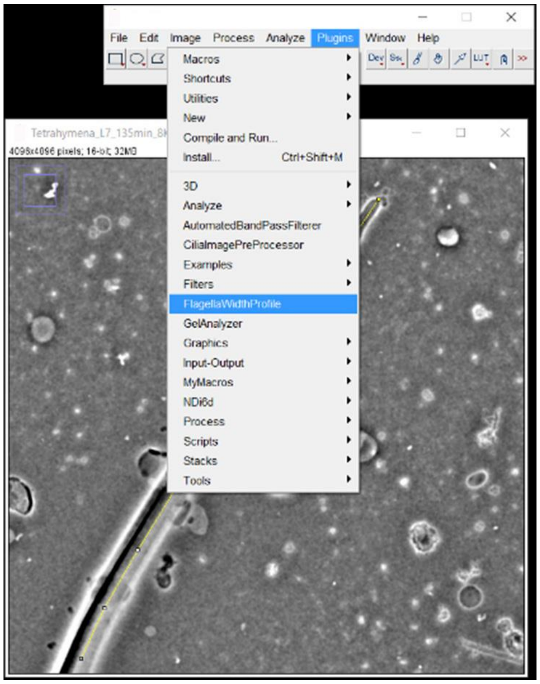

The tip region length was defined as the distance along the cilium's central axis from its absolute tip to where the slope of the best-fit curve was equal to 0.0524 (corresponding to a 1.5° slope on each side of the cilium) and the second derivative at that point was negative (indicating that the slope was decreasing at this point).

Workflow for Acquiring Width Profiles:

Images were first processed using the CiliaImagePreProcessor plugin for ImageJ. Before calling the plugin to process a batch of images, the images were stored in the same directory. Then, from ImageJ, the CiliaImagePreProcessor plugin was called. The plugin prompts the user to input parameters for image processing

using median and bandpass filters. We found that a median filter of 2.0 pixels and filtering large structures down to 100 pixels and filtering small structures up to 10 pixels were optimal for our images.

Once the appropriate parameters are entered, the user then is prompted to select the directory where the original images are located. Then, the user specifies the directory to store the new images, and the images are processed as a batch.

The FlagellaWidthProfile package can be implemented on individual images after they are processed. First, the user draws a polyline along the cilium's central axis using ImageJ's segmented line selection option, with a width of one pixel and no spline fit. A sample cilium is shown at right with a polyline drawn down its central axis. The polyline does not need to be positioned exactly in the center of cilium, but we found that the profiles were most accurate when it was drawn with the vertices closer to the central axis. Approximately ten vertices were usually placed per cilium, but more were required for cilia with high curvature.

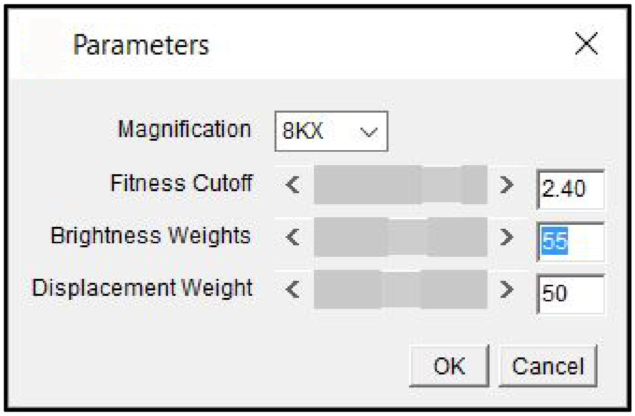

Once the polyline is drawn, FlagellaWidthProfile can be called from the "PlugIns" menu on ImageJ, as shown at right. This will call the package.

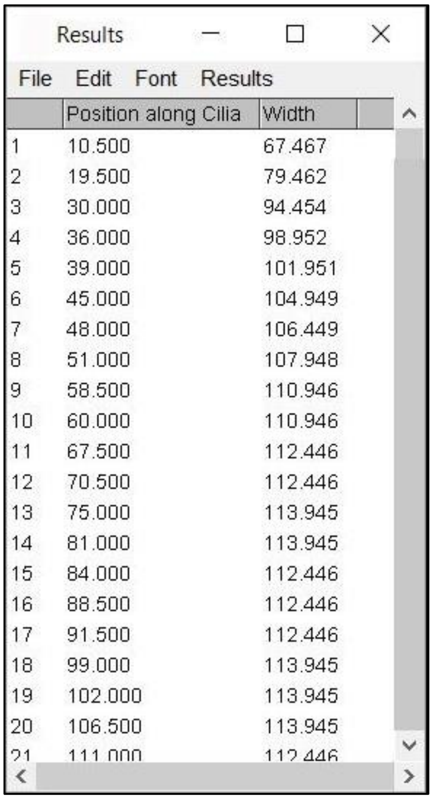

	Position along Cilia	Width
1	10.500	67.467
2	19.500	79.462
3	30.000	94.454
4	36.000	98.952
5	39.000	101.951
6	45.000	104.949
7	48.000	106.449
8	51.000	107.948
9	58.500	110.946
10	60.000	110.946
11	67.500	112.446
12	70.500	112.446
13	75.000	113.945
14	81.000	113.945
15	84.000	112.446
16	88.500	112.446
17	91.500	112.446
18	99.000	113.945
19	102.000	113.945
20	106.500	113.945
21	111.000	112.446

Once the package is called, a menu screen will appear that requires input from the user. The first parameter, "Magnification," specifies the magnification at which the image was acquired on the electron microscope. This parameter also has a "pixels" option if the microscope is uncalibrated or if the user desires the data in pixels instead of nanometers. The remaining three parameters are relevant to the fitness function portion of the width-determination algorithm. The "Fitness Cutoff" specifies an arbitrary fitness value below which peak-to-peak pairs are rejected. The "Brightness Weight" and "Displacement Weight" parameters respectively specify the relative contribution of the brightness of individual peaks and displacement of a particular pair of peaks from the polyline to the fitness of a peak-to-peak pair. The fitness cutoff typically was between 2.00 and 2.40, and the brightness and fitness cutoffs varied between 40 and 70. For each cilium, these values were manually adjusted to achieve an optimum width profile.

Once the plugin was executed, a scatterplot and a results table were displayed. Examples of each that result from the cilium shown are shown below. If the scatterplot was deemed to be of sufficient quality, the data from the results table was stored in a CSV file.

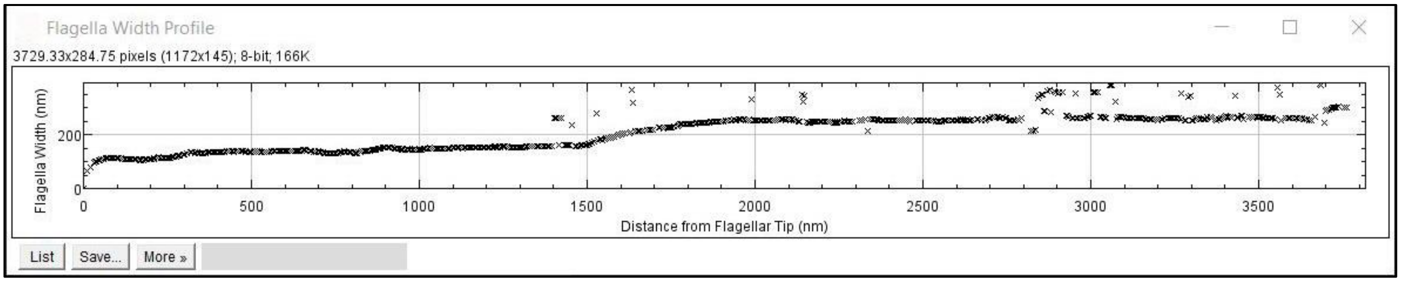

Supplement: Supplementary file 1 — Supplementary Materials [file 41598_2018_26111_MOESM1_ESM.pdf]
