# Supplementary material for: The Developmental Process of the Growing Motile Ciliary Tip Region
Source: Sci Rep. 2018 May 22;8:7977. doi: 10.1038/s41598-018-26111-2 (PMC5964098; doi:10.1038/s41598-018-26111-2)
Supplement: Supplementary file 2 — Custom tools for image analysis [file 41598_2018_26111_MOESM2_ESM.zip › cilium-tip-development_tools_installation_and_usage.pdf]

Installation and Execution Notes:

The text that follows is a detailed description of how to install and run the programs described above. The java source code for all files is included in the supplementary ZIP file.

In order to install the CiliaImagePreProcessor and FlagellaWidthProfile plugins in ImageJ, follow these steps:

1. Have a current version of ImageJ installed on your computer
2. Copy the CiliaPlugIn_.jar file from the ZIP to a location on your desktop
3. In ImageJ, click Plugins > Install…
4. Double click on the CiliaPlugIn_.jar file
5. Save the .jar file to your ImageJ plugins folder
6. Restart ImageJ. The two programs will be accessible in the "Plugins" menu under the tab “cilia”
7. Follow the steps outlined above to execute the programs on images of your cilia

In order to execute the GnuPlotInteracter and DerivativeCalculator programs, follow these steps:

1. Have a current version of GnuPlot installed on your computer in the “Programs File (x86)” directory (if you do not have a Windows computer, some minor modifications to the source code will be required)
2. Copy the GnuPlotInteracter.jar and DerivativeCalculator.jar files to a parent directory of your data
3. Once you have acquired several width profiles, run the GnuPlotInteracter.jar file in the command line using the command `java -jar GnuPlotInteracter.jar “ABSOLUTE_PATH_TO_FILES”` where the string parameter is the absolute path to the width profile comma-separated-value files. This program will output file names if it is successful
4. Navigate to the folder with the coefficients.txt file output from the last program
5. Execute the command `java –jar DerivativeCalculator.jar coefficients.txt`
6. The output will be the length of the ciliary tip region for each cilium. There may be more than one point on the curve that satisfies the conditions, and in this case, the value was determined very easily by inspection. It is recommended to always verify the measured tip region length with the original micrograph.
